# Photodynamic treatment of *Staphylococcus aureus* with non-iron hemin analogs in the presence of hydrogen peroxide†

**DOI:** 10.1039/d4md00148f

**Published:** 2024-05-03

**Authors:** Badhu Prashanthika Sivasubramaniam, Benjamin M. Washer, Yuichiro Watanabe, Kathryn E. Ragheb, J. Paul Robinson, Alexander Wei

**Affiliations:** a Department of Chemistry, Purdue University 560 Oval Drive West Lafayette IN 47907 USA alexwei@purdue.edu; b College of Veterinary Medicine, Purdue University 625 Harrison Street West Lafayette IN 47907 USA

## Abstract

Bacteria subjected to antiseptic or antibiotic stress often develop tolerance, a trait that can lead to permanent resistance. To determine whether photodynamic agents could be used to counter tolerance, we evaluated three non-iron hemin analogs (M-PpIX; M = Al, Ga, In) as targeted photosensitizers for antimicrobial photodynamic inactivation (aPDI) following exposure to sublethal H_2_O_2_. Al-PpIX is an active producer of ROS whereas Ga- and In-PpIX are more efficient at generating singlet oxygen. Al- and Ga-PpIX are highly potent aPDI agents against *S. aureus* and methicillin-resistant strains (MRSA) with antimicrobial activity (3 log reduction in colony-forming units) at nanomolar concentrations. The aPDI activities of Al- and Ga-PpIX against *S. aureus* were tested in the presence of 1 mM H_2_O_2_ added at different stages of growth. Bacteria exposed to H_2_O_2_ during log-phase growth were less susceptible to aPDI but bacteria treated with H_2_O_2_ in their postgrowth phase exhibited aPDI hypersensitivity, with no detectable colony growth after treatment with 15 nM Ga-PpIX.

## Abstract 2

The susceptibility of *S. aureus* to photodynamic inactivation can be greatly impacted by treatment with H_2_O_2_, depending on the timing of its exposure.

## Graphical abstract

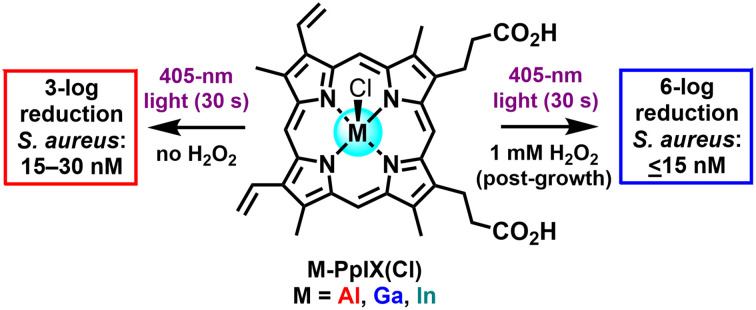

## Introduction

Antimicrobial resistance is a global health threat that continues to grow in severity.^1–3^ The rise of bacterial pathogens with multi-drug resistance, coupled with the sluggish development of novel antibiotics,^4,5^ is driving the need for innovative approaches to combat nosocomial contamination and infections.^6^ One possibility is to develop treatments that can override tolerance, an important but under-appreciated forerunner to more permanent forms of antimicrobial resistance.^7^ Bacterial subpopulations can develop tolerance if treatment times are insufficient; surviving cells can activate numerous repair genes that also increase the rate of mutations which lead to permanent resistance.^8^

To find ways of overcoming stress adaptation and tolerance, we considered antimicrobial photodynamic inactivation (aPDI), a topical, non-invasive treatment modality which has shown strong potential for combating drug-resistant strains of bacterial pathogens.^9,10^ Some reports suggest that aPDI agents have intriguing potential to increase microbial susceptibility to antibiotics, possibly due to higher membrane permeability.^11,12^ aPDI agents can also be used as light-activated antiseptics for decolonizing bacteria on dental implants, food packaging, and other surfaces that require sterilization.^13–15^ aPDI uses a photosensitizer (PS) to produce singlet oxygen (^1^O_2_) or reactive oxygen species (ROS) in a localized manner and offers a multimodal mechanism for killing bacteria, as opposed to antibiotics that act on a specific target. Importantly, the delivery of aPDI agents to bacterial colonies may be sufficient to produce a localized killing effect with limited adverse response from host cells and tissues.^9^

Many synthetic and natural dyes have been studied as photosensitizers.^16^ Tetrapyrrole-based macrocycles such as porphyrins, chlorins, and phthalocyanines are used extensively as aPDI agents, many of which are either clinically approved or currently under clinical trials for topical treatments such as cancer and acne.^17,18^ In particular, protoporphyrin IX (PpIX) and hematoporphyrin derivatives (HpD) have demonstrated good aPDI activity *in vivo*,^19,20^ and the polycation tetrakis(1-methyl-4-pyridinium)porphyrin (TMPyP) is highly active against multi-drug resistant bacteria as well as fungal species.^21^ However, many lack target specificity which contributes toward high PS loadings and subsequent phototoxicity.^19^ Current efforts to address the latter challenge include chemical modifications for targeted PS delivery, liposomal encapsulation, and conjugation to nanoparticles.^22^ Metal ions can also influence the PS properties of porphyrins and related tetrapyrroles by modulating intersystem crossing and ^1^O_2_ quantum yield.^23^

We^24,25^ and others^26,27^ have been investigating strategies for targeted aPDI based on the innate affinity of bacterial pathogens for hemin (Fe-PpIX), the oxidized form of heme. Many bacteria have hemin acquisition systems for the purpose of harvesting iron, an essential mineral for virulence and growth.^28^ Some express cell-surface receptors enabling the direct acquisition of hemin,^29,30^ whereas others deploy a more sophisticated system based on the release and diffusion of hemin-harvesting proteins (hemophores).^28^ Non-iron hemin analogs are thus promising candidates for targeted aPDI, as they can be delivered through a Trojan-horse mechanism.^31,32^ For example, replacing the Fe(iii) core with isoelectronic Ga(iii) results in a fluorescent derivative that can be delivered to a variety of bacteria *via* their hemin acquisition systems.^24^ Ga-PpIX is also a strong photosensitizer and can mediate aPDI of planktonic *S. aureus* and MRSA at nanomolar levels after just a few seconds of exposure to 405 nm light from an LED source. In contrast, Ga-PpIX has very low cytotoxicity with keratinocytes (HaCaT) and kidney cells (HEK293) maintaining 85–90% viability at 20 μM, the highest concentration tested.^24^

Multiple studies have shown that aPDI potency can be augmented in the presence of or pretreatment with H_2_O_2_, albeit with relatively high PS loadings and H_2_O_2_ levels.^33–40^ There are several postulates for the potentiation of aPDI by H_2_O_2_: (i) greater permeability of cell walls or membranes after H_2_O_2_ exposure,^37,41^ (ii) increased production of ROS and ^1^O_2_,^37^ and (iii) increased O_2_ levels from H_2_O_2_ disproportionation, possibly mediated by catalase-like activity.^40,42,43^ The latter effect may be helpful to combat bacterial infections in hypoxic or anaerobic environments.

**Fig. 1:**
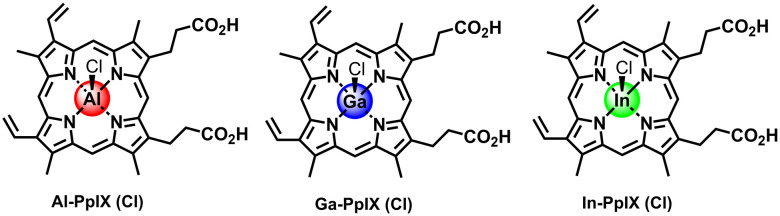
Chloride salts of non-iron hemin analogs (M-PpIX) with group 13 elements Al, Ga, and In.

In this work we compare the PS and aPDI activities of non-iron hemin analogs (M-PpIX) with group 13 metal ions, namely Al(iii), Ga(iii), and In(iii) (Fig. 1), and their potential for synergy with sublethal H_2_O_2_ (1 mM) to enhance antiseptic action against *S. aureus* and drug-resistant variants. The synergy can be remarkable under the right circumstances: H_2_O_2_-challenged strains of *S. aureus* can be reduced below the limit of detection at loadings as low as 15 nM M-PpIX, depending on the PS type as well as conditioning methods used.

## Results and discussion

### Chemical and photophysical characterization of M-PpIX

**Fig. 2:**
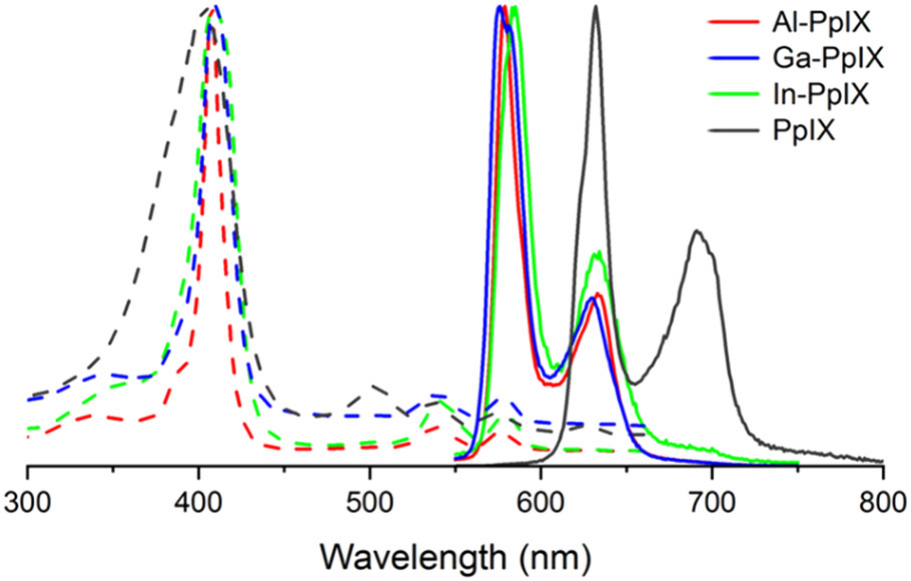
Normalized absorption (dashed curves) and luminescence spectra (solid curves) of M-PpIX(Cl) (M = Al, Ga, and In) and PpIX in 8 μM in DMSO.

Characterization of Ga-PpIX(Cl) has been described previously^24^ and is included for analysis of periodic trends. The absorption spectra of all M-PpIX(Cl) salts (8 μM in DMSO) exhibit intense Soret bands at 405 nm, similar to PpIX but with narrower linewidths, and lack two peaks in the Q band region at 500 and 625 nm, characteristic of porphyrin–metal ion complexes (Fig. 2).^44^ Photoluminescence spectroscopy reveals a large blueshift in M-PpIX emission (45–55 nm relative to PpIX), with a primary emission band at 575–585 nm and a secondary band at 628–635 nm (Table 1). The fluorescence quantum yields (*Φ*_FL_) for 405 nm excitation of Al-PpIX, Ga-PpIX and In-PpIX are 12%, 6.3% and 1% respectively, the latter diminished by the faster intersystem crossing rate due to spin–orbit coupling (heavy atom effect).^45^ On the other hand, ^1^O_2_ quantum yields for M-PpIX are enhanced by the heavy-atom effect: *Φ*_SO_ for Ga- and In-PpIX are 0.45–0.46, whereas that for Al-PpIX is only 0.12.

**Table 1:** Photophysical properties of M-PpIX(Cl) (M = Al, Ga, In)^a^

M	*λ*_abs_ (nm)	*λ*_em_ b (nm)	*Φ*_FL_ b (%)	*Φ*_SO_ c (%)
Al(iii)	405, 540, 576	585, 641	12.0	12
Ga(iii)	405, 541, 578	585, 640	6.3	45
In(iii)	405, 541, 579	586, 636	1.0	46

a Studies in 8 μM in DMSO.

b Excitation at 405 nm.

c Relative to TMPyP (*Φ*_SO_ 75%).

### Flow cytometry analysis of M-PpIX uptake by *S. aureus*

Our lab has determined previously that active cultures of *S. aureus* acquire Ga-PpIX rapidly (within seconds) and with high affinity,^24^ which we attribute to the expression of cell-surface hemin receptors such as Isd proteins.^31,46^ The fluorescence of Ga-PpIX (and lack of fluorescence from Fe-PpIX) enabled us to establish CSHR specificity by a hemin competition experiment.^24^ With regard to the possible identity of CSHRs, X-ray crystal structures have been reported of Ga- and In-PpIX bound to the NEAT3 domain of IsdH receptors expressed by *S. aureus*.^47,48^

**Fig. 3:**
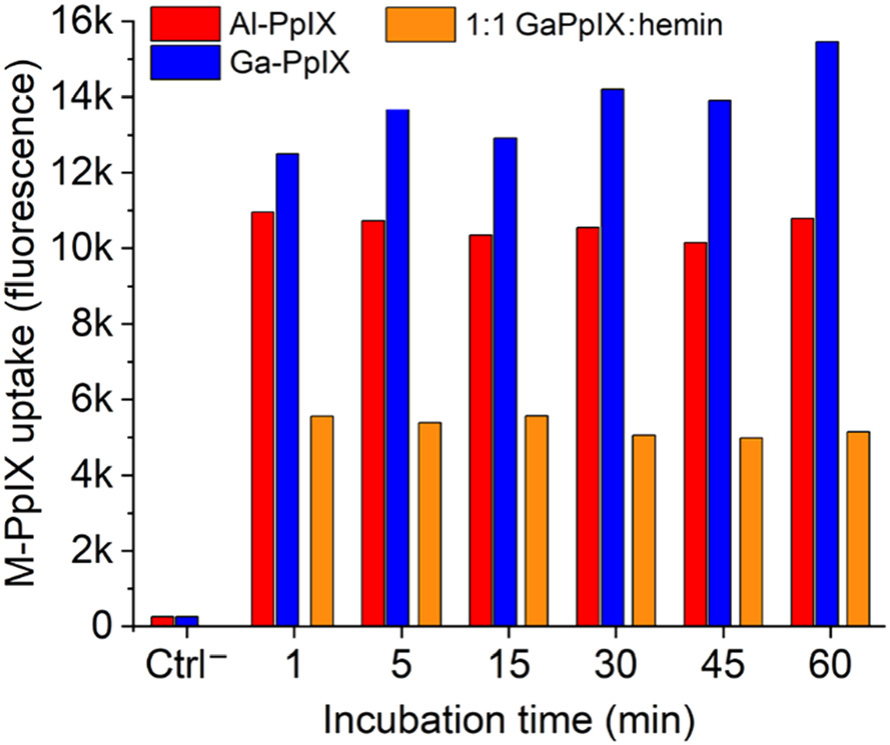
Histogram of flow cytometry (FC) data for *S. aureus* (PCI 1203) cultured in Fe-deficient media, following treatment with 10 μM M-PpIX, where M = Al (red) or Ga (blue). Specificity for M-PpIX uptake by *S. aureus* established by competition assay using a 1 : 1 Ga-PpIX : hemin mixture (orange). All bacteria were fixed after M-PpIX treatment and before FC injection; see ESI† for original FC data.

The strong fluorescence of Al- and Ga-PpIX also make them ideal candidates for using flow cytometry (FC) to study their time-dependent uptake. Bacterial suspensions of *S. aureus* cultured in iron-deficient conditions were treated with Al- or Ga-PpIX diluted in PBS (final concentration 10 μM) and incubated at room temperature between 60 s and 60 min, then fixed with 4% paraformaldehyde and subjected directly to FC. In both cases, fluorescence signals were already at their maximum intensity from the initial injection and remained little unchanged over time (Fig. 3 and S1, ESI†). This indicates M-PpIX uptake to be a diffusion-controlled process with low susceptibility to efflux pump activity, which is known to play a role in heme homeostasis.^49^ We also performed a competitive uptake assay using a 1 : 1 ratio of M-PpIX and hemin, which strongly impacted signal intensity and confirmed the results of our previous study by fluorescence imaging.^24^ We note that *S. aureus* cultured under iron-replete conditions responded less avidly to M-PpIX, implying that CSHR expression is upregulated upon iron deprivation.

The diffusion-controlled uptake of M-PpIX led us to postulate that CHSR expression alone should be sufficient for targeted delivery. To test this, *S. aureus* cultures were fixed prior to treatment with M-PpIX and subjected to FC analysis at various time points (Fig. S2, ESI†). Remarkably, no apparent differences in fluorescence intensity or uptake over time were observed, with results essentially identical to those shown in Fig. 3. We thus consider M-PpIX uptake to be an energy-independent process mediated purely by cognate CSHRs presented on the bacterial surface.

### Antibacterial and aPDI activity of M-PpIX

**Fig. 4:**
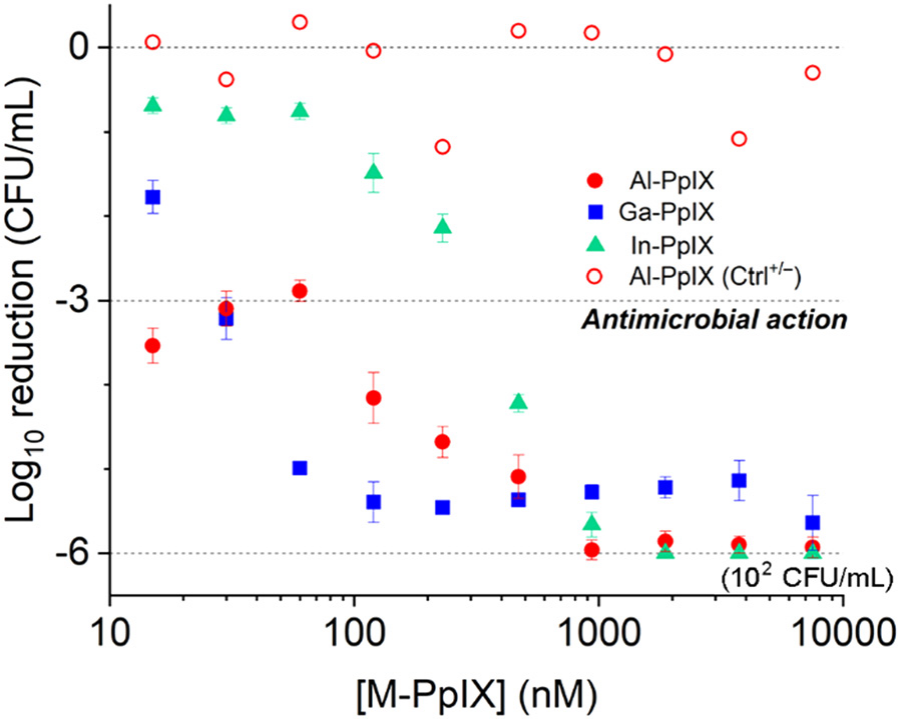
Antimicrobial photodynamic inactivation (aPDI) of *S. aureus* (PC 1203) by M-PpIX, with 30 s exposure to 405 nm light (4.2 J cm^−2^). Data for antimicrobial activity of Al-PpIX without light (Ctrl^+/−^) included for comparison.

Antimicrobial activities of M-PpIX derivatives were evaluated using planktonic *S. aureus* plus four clinical isolates of MRSA cultured in iron-deficient media (Fig. 4 and Table 2). In the absence of light, the minimum inhibitory concentration (MIC) values for all combinations of M-PpIX and *S. aureus* was 30 μM; with 30 seconds of 405 nm irradiation, the MIC values were lowered to 15 μM. The full potency of aPDI was established by quantifying colony log reduction following irradiation, with 3 log reductions in the nanomolar range. Al- and Ga-PpIX were most active against *S. aureus* with 3 log reduction values as low as 0.015 μM and 0.03 μM respectively; aPDI with In-PpIX was much less potent (0.24 μM) although still comparable to TMPyP, a leading PS.^24^ Little to no reduction in *S. aureus* populations was observed in the absence of 405 nm light (Ctrl^+/−^) or M-PpIX (Ctrl^−/+^). Interestingly, Al-PpIX was less potent against three of the four MRSA strains whereas the aPDI activity of Ga-PpIX was essentially constant. This indicates that (i) the less susceptible MRSA strains have active mechanisms for quenching ROS, and (ii) none of the bacteria have defense mechanisms to counter ^1^O_2_, which has an aqueous lifetime of just a few microseconds.^50^

**Table 2:** Antimicrobial activities of M-PpIX against *S. aureus* and MRSA^a^

Bacterial strain	Activity	Al-PpIX (μM)	Ga-PpIX (μM)	In-PpIX (μM)
*S. aureus* (PCI 1203)	MIC (dark)^b^	30	30	30
	MIC (irrad)^b^^,^^c^	15	15	15
	aPDI^d^	0.015	0.03	0.23
MRSA, USA 300	aPDI	0.015	0.03	0.03
(NRS 383)				
MRSA, USA 300	aPDI	0.06	0.03	0.12
(NRS 385)				
MRSA, USA 300	aPDI	0.23	0.015	0.23
(NRS 386)				
MRSA, USA 300	aPDI	0.06	0.03	0.46
(NRS 387)				

a Standard conditions: 10^8^ CFU mL^−1^ in Fe-deficient media prior to dilution with PBS.

b MIC: data obtained 16 h after M-PpIX treatment.

c aPDI: 30 s irrad. with 405 nm LED source (4.2 J cm^−2^).

d Minimum concentrations for 3 log reduction.

### aPDI activity of M-PpIX against H_2_O_2_-challenged *S. aureus*

The prevalence of *S. aureus* and MRSA in hospital-associated infections is supported by their ability to adapt and survive adverse conditions during routine sterilizations. For example, *S. aureus* employs a variety of defense mechanisms to combat acute oxidative stress such as biofilm formation and the overexpression of catalase, superoxide dismutase, and staphyloxanthin.^51^ For longer periods of stress, bacteria can transform into small-colony variants (SCVs) with high tolerance to antibiotics and H_2_O_2_.^52,53^ In this work, we investigated the aPDI efficacy of Al- and Ga-PpIX against *S. aureus* following their adaptation to sublethal levels of H_2_O_2_.

**Scheme 1:**
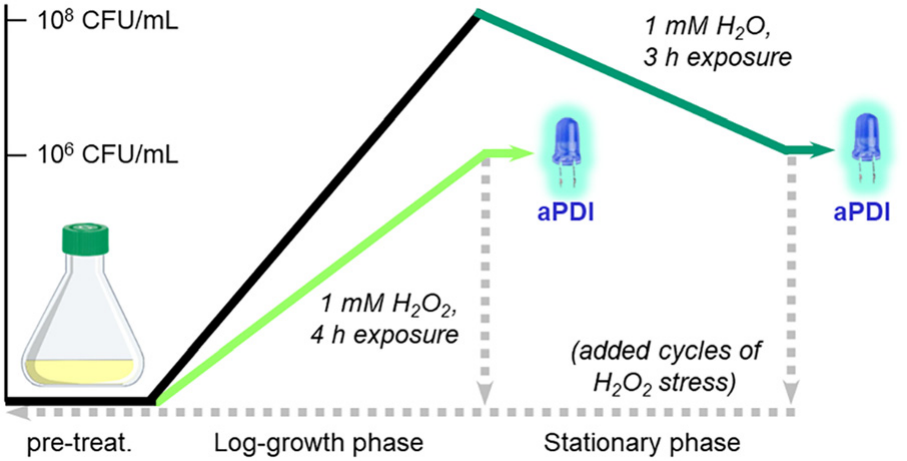
*S. aureus* challenged with 1 mM H_2_O_2_ during log-growth phase (light green) or stationary phase (dark green), prior to aPDI with initial population of 10^6^ CFU mL^−1^. Bacteria were also harvested (dashed lines) and resubjected to 1 mM H_2_O_2_ after overnight incubation up to 4 times, followed by aPDI to assess adaptation to oxidative stress.

The experimental design for this study is summarized in Scheme 1. *S. aureus* (PCI 1203) cultured in Fe-deficient media were challenged with 1 mM H_2_O_2_ at either an early stage (4 h during log-growth phase) or a late stage (3 h during stationary phase) followed by aPDI with Al- or Ga-PpIX (cycle 1); the initial population in both cases was 10^6^ CFU mL^−1^. In addition, cultures of H_2_O_2_-challenged bacteria were harvested then resubjected to 1 mM H_2_O_2_ during early- or late-stage growth (cycles 2–4) to determine adaptation to oxidative stress over time.

**Fig. 5:**
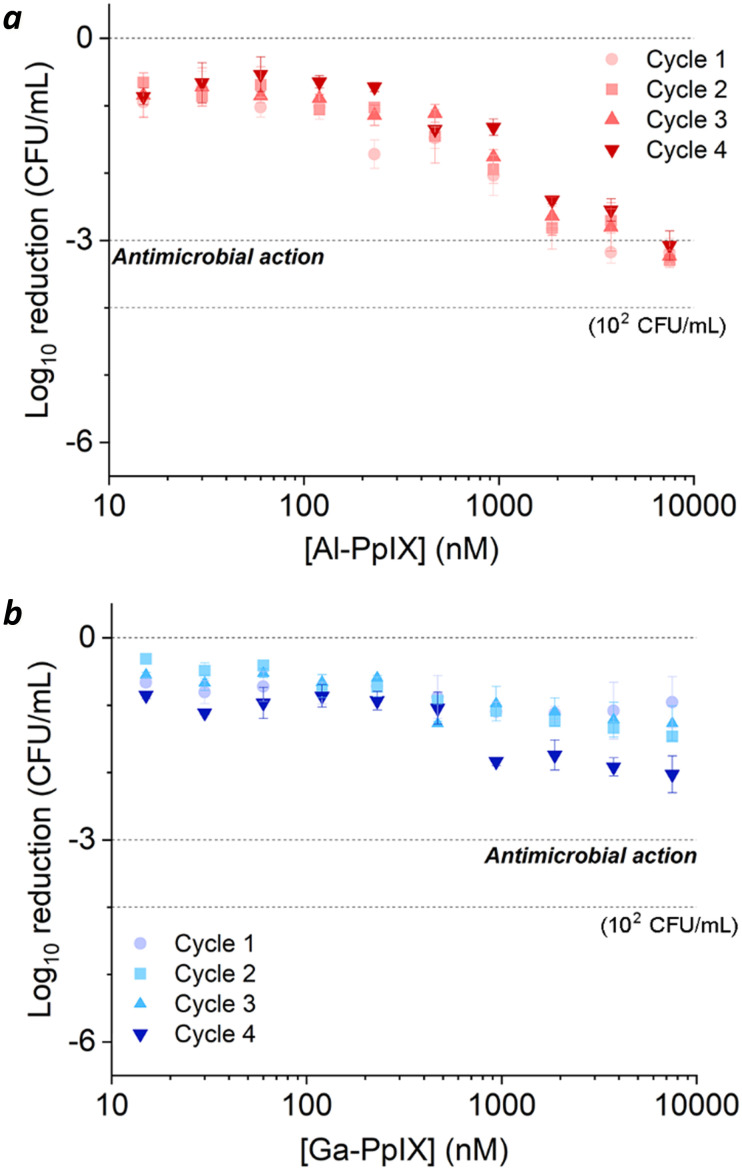
aPDI of *S. aureus* challenged by 1 mM H_2_O_2_ during the log-growth phase, repeated over 4 cycles. (a) Al-PpIX; (b) Ga-PpIX. Studies were conducted with 30 s exposure to 405 nm light (4.2 J cm^−2^) using an initial population of 10^6^ CFU mL^−1^.

Bacteria challenged with 1 mM H_2_O_2_ during early-stage growth had low sensitivity to aPDI. A 3 log reduction could only be achieved with Al-PpIX above 4 μM, a tolerance that persisted over several growth cycles (Fig. 5a). Low sensitivity to ROS is expected as antioxidant enzymes are upregulated in response to oxidative stress;^54^ for example, aPDI of *E. coli* and *E. faecalis* can induce the upregulation of oxidative stress genes such as dps, hypR, and soxRS^55,56^ as well as general stress markers such as *σ*-factors,^55,57^ and aPDI treatment of *S. aureus* with PpIX can increase the expression of superoxide dismutase.^58^

Surprisingly, H_2_O_2_-challenged bacteria were also highly tolerant to Ga-PpIX-mediated aPDI, which did not produce a 3 log reduction even at 10 μM (Fig. 5b). To our knowledge, there are no reports linking known oxidative stress responses with protection against ^1^O_2_. To explain the low sensitivity to ^1^O_2_ we considered the possibility that H_2_O_2_ exposure lowered CSHR expression and M-PpIX avidity, however FC analysis of H_2_O_2_-treated *S. aureus* with Ga-PpIX did not reveal any differences in time-dependent uptake (Fig. S3, ESI†). Further studies are needed to elucidate the apparently low susceptibility of these cultures to Ga-PpIX-mediated aPDI.

**Fig. 6:**
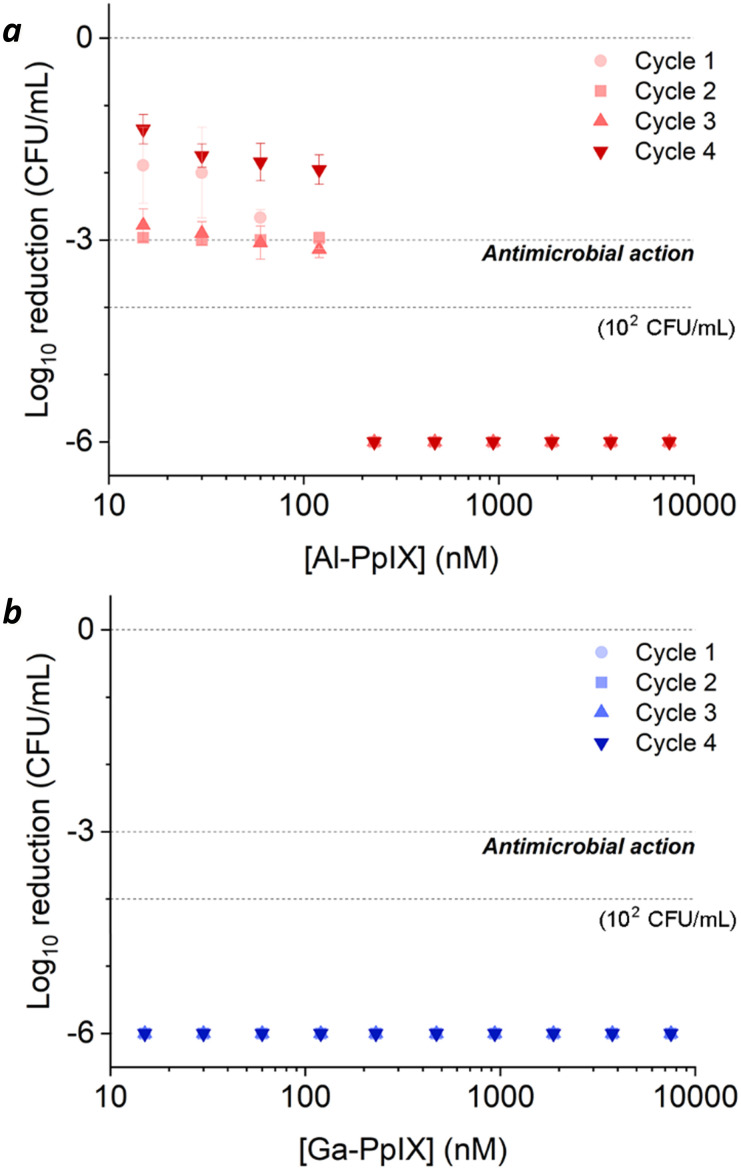
aPDI of *S. aureus* challenged by 1 mM H_2_O_2_ after the log-growth phase, repeated over 4 cycles. (a) Al-PpIX; (b) Ga-PpIX. Studies were conducted with 30 s exposure to 405 nm light (4.2 J cm^−2^) using an initial population of 10^6^ CFU mL^−1^.

The effects of aPDI were strikingly different for *S. aureus* cultures challenged with H_2_O_2_ after their log-growth phase (Fig. 6). A 3 h exposure to 1 mM H_2_O_2_ reduced the bacterial population from 10^8^ to 10^6^ CFU mL^−1^, which was insufficient for antimicrobial activity by itself but caused surviving populations to become hypersensitive to aPDI. Cultures experienced a 3 log reduction when treated with 0.015 μM Al-PpIX and below the limit of detection (∼6 log reduction) was achieved at 0.24 μM, several fold lower than that for naïve *S. aureus* (Table 3). Most impressively, Ga-PpIX had outstanding potency with reduction below the limit of detection achieved at 0.015 μM, the lowest PS concentration tested in this study.

**Table 3:** aPDI activities of M-PpIX against *S. aureus* (PCI 1203) in 1 mM H_2_O_2_

Condition	aPDI^a^	Al-PpIX (μM)	Ga-PpIX (μM)
Standard^b^ (no H_2_O_2_ added)	3 log	0.015	0.03
	6 log	0.90	0.24^c^
1 mM H_2_O_2_, growth phase^d^	3 log	3.76^e^	*n/o*
	6 log^f^	*n/o*	*n/o*
1 mM H_2_O_2_, stationary phase^d^	3 log	0.015^g^	**<0.015**
	6 log^f^	0.24	**≤0.015**

a Reduction in CFU mL^−1^ from initial population.

b Standard conditions: 7 h in Fe-deficient media (4 h growth, 3 h lag); 10^8^ CFU mL^−1^ prior to dilution with PBS. aPDI: 30 s irrad. with 405 nm LED source (4.2 J cm^−2^).

c 5 log reduction in CFU mL^−1^.

d Initial 2 log reduction not included in aPDI.

e Cycle 1.

f Below 100 CFU mL^−1^.

g Cycle 2. *n/o* = not observed.

The dramatically higher aPDI potency following post-growth H_2_O_2_ exposure may be due to an increase in either catalase activity or cell wall permeability. The latter effect has been noted in both microbial and mammalian cells and is thought to be responsible for increased drug or PS uptake.^12,36,40,41^ Moreover, bacterial cultures are less adept at responding to stress in the stationary phase compared with the log-growth phase, when survival genes can be actively selected.^59,60^ With regard to hypersensitivity to Ga-PpIX, we postulate that H_2_O_2_ induces the upregulation of catalase which increases the local concentration of O_2_. Photodynamic inactivation is much more potent in hyperoxic environments, which can be triggered by a variety of catalysts in the presence of H_2_O_2_.^42,43,61^

The relative susceptibility of H_2_O_2_-challenged *S. aureus* can evolve with the number of exposures and growth cycles. For cultures treated with 1 mM H_2_O_2_ during log-phase growth, tolerance to aPDI remains high but a gradual increase in sensitivity to Ga-PpIX-mediated aPDI can be observed (Fig. 5b). Repeated H_2_O_2_ exposures may be selecting for subpopulations that produce high levels of catalase, resulting in higher local O_2_ concentrations and greater sensitivity to ^1^O_2_. On the other hand, cultures treated with 1 mM H_2_O_2_ postgrowth exhibit decreased sensitivity to Al-PpIX-mediated aPDI at later cycles (Fig. 6a), which can be ascribed to an adaptation against ROS stress. H_2_O_2_ is also known to trigger a bacterial SOS response that activates the upregulation of DNA repair enzymes,^53,62^ and this may also contribute toward aPDI tolerance over time. We note that while Al-PpIX loses its efficacy at low concentrations after repeated H_2_O_2_ exposures, its rate of ROS production above 0.1 μM is sufficient to overcome tolerance and eradicate H_2_O_2_-adapted bacteria.

## Conclusions

Non-iron hemin analogs with group 13 atoms (Al-, Ga-, and In-PpIX) are excellent photosensitizers for targeted aPDI against *S. aureus* and MRSA strains. Fluorescent Al-PpIX produces mostly ROS whereas Ga- and InPpIX are more efficient at producing singlet oxygen. Al- and Ga-PpIX are captured by *S. aureus* within seconds and can mediate aPDI at 405 nm excitation with 3 log reductions at 15–30 nM, but their potencies are greatly reduced against bacteria grown in the presence of 1 mM H_2_O_2_. On the other hand, *S. aureus* exposed to H_2_O_2_ in their postgrowth phase are hypersensitized to aPDI, with 6 log reduction at 240 nM for Al-PpIX and 15 nM for Ga-PpIX. The stark contrast in the aPDI sensitivity of *S. aureus* challenged by H_2_O_2_ at different stages in its lifecycle underscores a complex relationship between stress response and adaptation, revealing opportunities to exploit potential weaknesses in bacterial defense mechanisms.

## Experimental

### General

Hemin chloride and all reagents were obtained from commercial sources and used as received. The preparation of PpIX and the Cl salts of Al-PpIX, Ga-PpIX, and In-PpIX have been described in the literature previously and were reproduced with little modification.^24,63–65^ Absorption spectra of M-PpIX were collected on a Varian Cary50 spectrometer using a 1 cm quartz cuvette; luminescence spectra and fluorescence quantum yields (*Φ*_FL_) were measured using an Edinburgh Instruments FLS 980 spectrometer with an integrating sphere accessory. EPR studies were performed using a Bruker EMX X-band spectrometer operating at 9.5 GHz and 5.02 mW with a field modulation amplitude of 5 g at 100 kHz and used to estimate *Φ*_SO_ with 405 nm excitation and TMPyP as a reference compound, based on the EPR method reported by Nakamura *et al.*^66^ EPR data analysis is provided in ESI† (Fig. S4).

### Microbiological cultures

*S. aureus* PC 1203 was obtained from the American Type Culture Collection (ATCC 10537) and cultured at 37 °C in standard tryptic soy (TS) broth (Fe-replete conditions), and also in media containing 3 mM 2,2′-bipyridine (Fe-deficient conditions). Clinical isolates of MRSA were cultured in a similar fashion.^24,25^ Cultures were typically incubated for 16 h then diluted to achieve an optical density of 0.5 at 600 nm (10^8^ CFU mL^−1^). Bacterial counts were determined by plating serial dilutions on agar dishes and incubating at 37 °C for 16 h.

### Bacterial uptake assays using flow cytometry

FC studies were performed using a Cytoflex instrument (*λ*_ex_ = 488 nm; 585/42 nm emission filter). Stock solutions of Al- or Ga-PpIX were prepared in DMSO (1 mM) and diluted in phosphate buffered saline (PBS, pH 7.4) just prior to use. Bacterial suspensions (10^8^ CFU in 0.5 mL) were incubated with M-PpIX (10 μM) for specified periods between 10 s and 30 min, then fixed with 0.5 mL of 4% paraformaldehyde and subjected to FC analysis without further washing at a flow rate of 10 μL min^−1^. Bacterial cells (*N* = 10^5^) were gated by defining a region of interest (ROI) based on forward and side scattering (FSC and SSC) parameters (Fig. S1, ESI†). For experiments involving labeling by inactivated samples, fixed bacteria were collected by centrifugation and redispersed in PBS before treatment with Ga-PpIX. Samples were incubated between 0.5 and 30 min, then analyzed by flow cytometry without further washing.

### Antimicrobial activities

MIC values were obtained using the broth microdilution method.^67^ Bacterial suspensions (final concentration 5 × 10^6^ CFU mL^−1^) were diluted with one volume of M-PpIX solution with twofold serial dilution in microwells and incubated at 37 °C for 16 h. MIC values were determined by visual turbidity and confirmed in some cases by plating surviving bacteria on agar plates.

aPDI assays were performed on planktonic *S. aureus* and MRSA (10^6^–10^8^ CFU mL^−1^) using 96-well plates irradiated by a 405 nm LED array (Rainbow Technology Systems, 140 mW cm^−2^). In a typical study, bacteria were cultured in standard TS broth then sub-cultured under Fe-deficient conditions prior to serial dilution in microtiter plates (0.2 mL per well). Bacterial suspensions were treated with 100 μL of M-PpIX with final concentrations ranging from 0.015 to 60 μM, followed immediately with a 30 s exposure to 405 nm light (4.2 mJ cm^−2^). Irradiated bacteria were then plated onto agar plates using serial tenfold dilutions; controls included bacteria irradiated without M-PpIX (Ctrl^−/+^) and bacteria with M-PpIX but no irradiation (Ctrl^+/−^). Bacterial counts were determined by the drop-plate method using TS-agar plates;^68^ aPDI susceptibilities were quantified by subtracting final log counts from initial values (log 8 or log 6).

### aPDI studies with H_2_O_2_-treated bacteria

H_2_O_2_-tolerant (aPDI-insensitive) cultures were induced by cultivating *S. aureus* in standard media at 37 °C for 16 h. Optical density was adjusted to 0.5 followed by 100-fold dilution in Fe-deficient media containing 1 mM H_2_O_2_ and incubation at 37 °C for 4 h (log-growth phase; Scheme 1). This typically yielded bacterial densities close to 10^6^ CFU mL^−1^; aPDI assays were then performed using the procedure described above. Repeat growth cycles were performed by transferring 50 μL of the H_2_O_2_-treated culture into 5 mL of fresh Fe-replete media and incubating at 37 °C for 16 h. Optical density was adjusted to 0.5 followed by 100-fold dilution in Fe-deficient media with 1 mM H_2_O_2_ as above.

H_2_O_2_-challenged (aPDI-sensitive) cultures were induced by first cultivating *S. aureus* in Fe-deficient media until 10^8^ CFU mL^−1^ was achieved, followed by post-growth treatment with sublethal H_2_O_2_ (final concentration 1 mM) and incubation at 37 °C for 4 h (stationary phase, Scheme 1) with final bacterial densities close to 10^6^ CFU mL^−1^. aPDI assays and repeat growth cycles were performed using the procedures described above.

## Author contributions

B. P. S. conducted all microbiological and aPDI studies; B. M. W. and B. P. S. synthesized M-PpIX derivatives and performed EPR analysis of *Φ*_SO_; Y. W. collected optical properties and *Φ*_FL_; B. P. S., K. E. R. and J. P. R. conducted FC analysis; B. P. S. and A. W. designed the research study and wrote the manuscript.

## Conflicts of interest

There are no conflicts to declare.

## Supplementary Material

MD-015-D4MD00148F-s001
