# Supplementary material for: Photodynamic treatment of Staphylococcus aureus with non-iron hemin analogs in the presence of hydrogen peroxide
Source: RSC Med Chem. 2024 May 3;15(6):2138–45. doi: 10.1039/d4md00148f (PMC11187572; doi:10.1039/d4md00148f)
Supplement: MD-015-D4MD00148F-s001 [file MD-015-D4MD00148F-s001.pdf]

Electronic Supplementary Material (ESI) for RSC Medicinal Chemistry.
This journal is © The Royal Society of Chemistry 2024

B. Sivasubramaniam, B. M. Washer, Y. Watanabe, K. Ragheb, J. P. Robinson, A. Wei

Photodynamic treatment of *Staphylococcus aureus* with non-iron hemin analogs in the presence of hydrogen peroxide

Synthesis of M-PpIX derivatives S-1
One- and two-dimensional plots of representative flow cytometry data (Fig. S1)...... S-2
Flow cytometry data for Ga-PpIX uptake by fixed *S. aureus* (Fig. S2) S-3
Flow cytometry data for Ga-PpIX uptake by *aureus* exposed to H_2O_2 (Fig. S3)........ S-3
EPR data for estimating Φ_{SO} of M-PpIX with 405-nm excitation (Fig. S4)............... S-4

Synthesis of M-PpIX derivatives: Al-, Ga-, and In-PpIX chloride were prepared from protoporphyrin IX (PpIX) using previously published protocols,[24,63] with minor modifications in the case of Al-PpIX.[64,65]

In a typical reaction, PpIX dimethyl ester was prepared by dissolving PpIX (500 mg) in a 5% H_2SO_4 solution in methanol (11 mL) and stirred for 18 h at room temperature with protection from light, then neutralized with saturated aqueous NH_4Cl and extracted three times with CH_2Cl_2. The organic layer was washed with water and dried over Na_2SO_4, then concentrated under vacuum to yield a black solid in 90% yield. Al-PpIX was prepared by dissolving PpIX dimethyl ester (100 mg) in distilled pyridine (4 mL) in a glass microwave tube followed by addition of excess $AlCl_3$ (250 mg). The reaction mixture was heated to 140 °C under microwave conditions (45 W) for 60 min. Al-PpIX dimethyl ester was extracted by removing the supernatant, washing the residual solid with pyridine (3 × 3 mL), and concentrating the extract to dryness. Saponification to Al-PpIX was achieved by dissolving the crude dimethyl ester in 10 mL of 1,4-dioxane and 1 mL of 2 wt% NaOH and heating for 3 h under reflux conditions. The mixture was cooled to room temperature, neutralized with 1 M HCl then adjusted to pH 4.5, and concentrated to dryness. The solid containing Al-PpIX was suspended in methanol, filtered, then extracted three times with chloroform. The extracts were concentrated and dried under vacuum to yield Al-PpIX as a dark-red solid in 20% yield.

Flow cytometry data

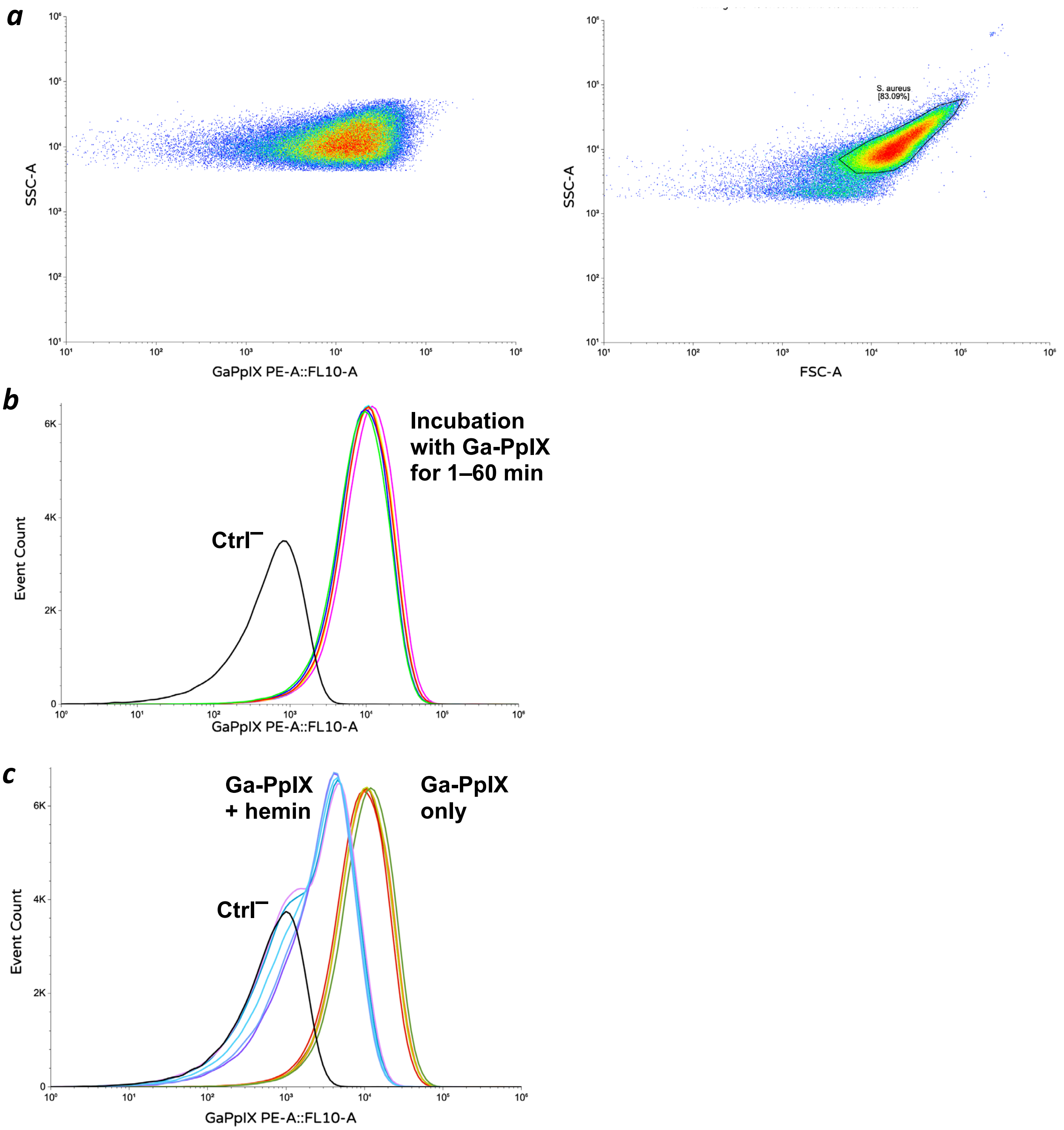

Figure S1. (a) Fluorescence and gated 2D contour plots of *S. aureus* (PCI 1203) treated with 10 µM Ga-PpIX for 60 min. Cultures were fixed with 4% paraformaldehyde then subjected immediately to FC analysis. (b) 1D FC plot with log fluorescence intensity (x-axis); peak values replotted with log values (y-axis) in Fig. 3a. (c) 1D FC plot with log fluorescent intensity (x-axis) of *S. aureus* incubated with 10 µM Ga-PpIX plus 10 µM hemin for 1–60 min; peak values replotted with linear values (y-axis) in Fig. 3b.

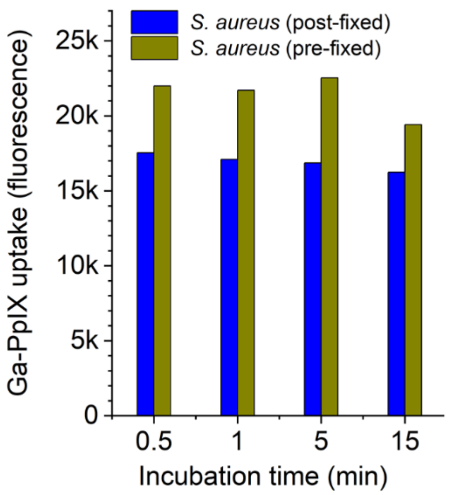

Figure S2. Histogram of FC data for *S. aureus* treated with 10 μM Ga-PpIX for up to 15 min with post-fixing (blue; cf. Figures 3 and S1) versus pre-fixed *S. aureus* treated under identical conditions (green).

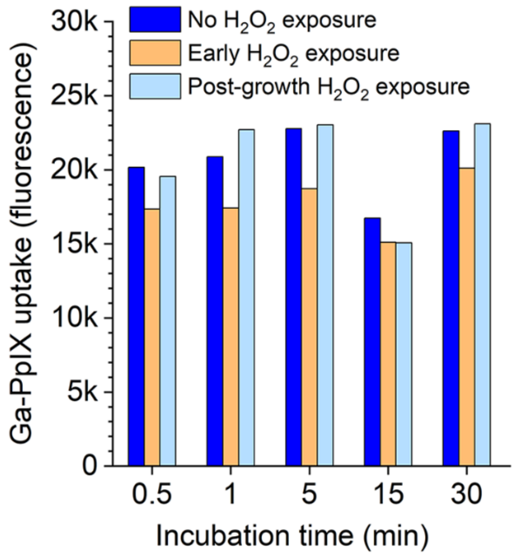

Figure S3. Histogram of FC data for *S. aureus* exposed to 1 mM H_2O_2 for 4 h during log-growth phase (early H_2O_2 exposure; orange) or 3 h during post-growth phase (late H_2O_2 exposure; light blue) followed by treatment with 10 μM Ga-PpIX for up to 30 min. Cultures were fixed with 4% paraformaldehyde then subjected immediately to FC analysis. *S. aureus* cultured in the absence of H_2O_2 were included as a control (no H_2O_2 exposure; dark blue).

EPR data

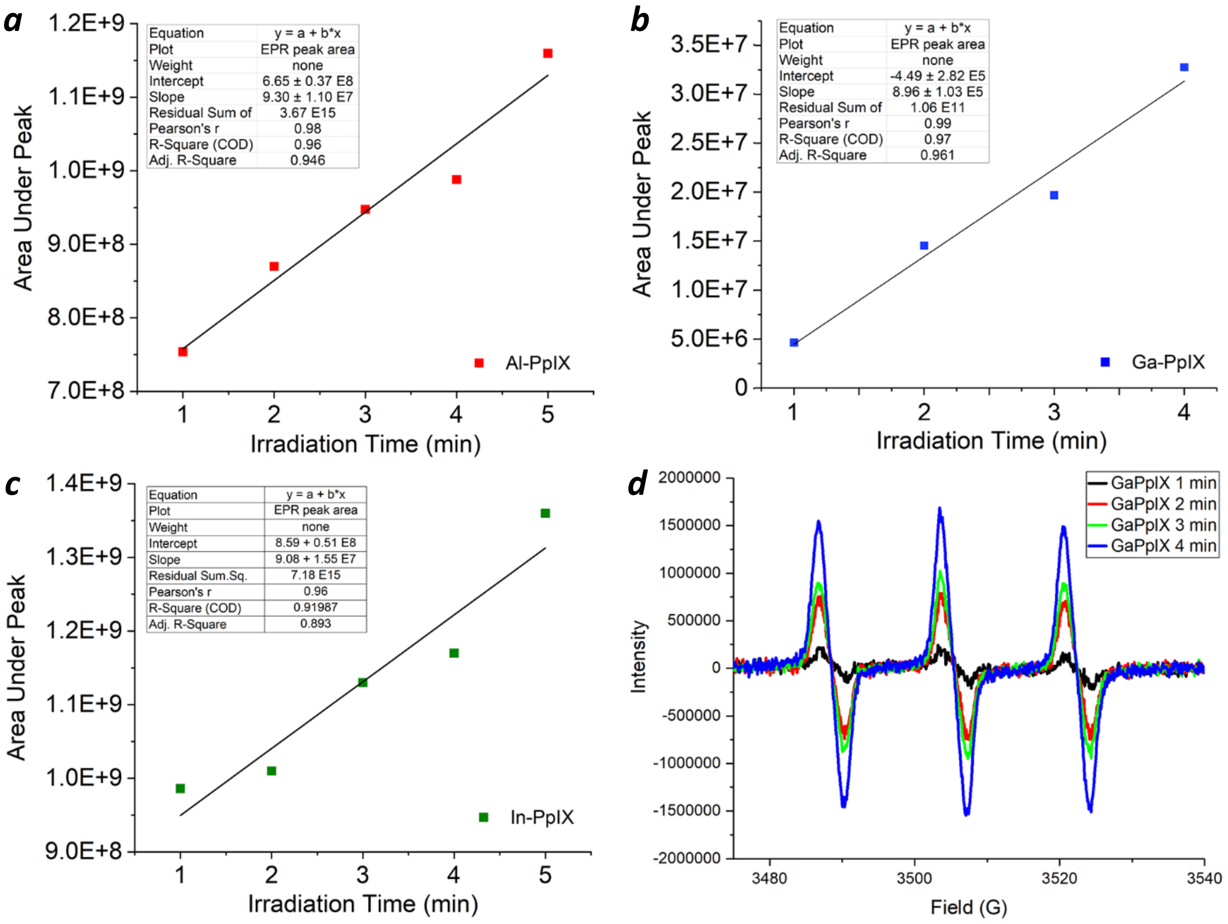

Figure S4. (a–c) Standard curves based on peak area integration of EPR signals from 4-hydroxy-TEMPO produced by M-PpIX as a measure of $^1O_2$ generation: (a) M = Al; (b) M = Ga, (c) M = In. Rates of EPR signal increase were compared against those generated by TMPyP (Φ_{SO} = 0.75) to determine SOQY. (d) EPR signals of 4-hydroxy-TEMPO produced by Ga-PpIX after 1–4 min irradiation at 405 nm, using the method reported in Ref. 66. EPR data for Figures (b) and (d) originally reported in Ref. 24.
